# IGF1 Is a Common Target Gene of Ewing's Sarcoma Fusion Proteins in Mesenchymal Progenitor Cells

**DOI:** 10.1371/journal.pone.0002634

**Published:** 2008-07-09

**Authors:** Luisa Cironi, Nicolò Riggi, Paolo Provero, Natalie Wolf, Mario-Luca Suvà, Domizio Suvà, Vincent Kindler, Ivan Stamenkovic

**Affiliations:** 1 Division of Experimental Pathology, Institute of Pathology CHUV, University of Lausanne, Lausanne, Switzerland; 2 Department of Biology Genetics and Biochemistry, University of Turin, Turin, Italy; 3 Department of Orthopedics, University Hospital, University of Geneva, Geneva, Switzerland; Deutsches Krebsforschungszentrum, Germany

## Abstract

**Background:**

The EWS-FLI-1 fusion protein is associated with 85–90% of Ewing's sarcoma family tumors (ESFT), the remaining 10–15% of cases expressing chimeric genes encoding EWS or FUS fused to one of several ets transcription factor family members, including ERG-1, FEV, ETV1 and ETV6. ESFT are dependent on insulin-like growth factor-1 (IGF-1) for growth and survival and recent evidence suggests that mesenchymal progenitor/stem cells constitute a candidate ESFT origin.

**Methodology/Principal Findings:**

To address the functional relatedness between ESFT-associated fusion proteins, we compared mouse progenitor cell (MPC) permissiveness for EWS-FLI-1, EWS-ERG and FUS-ERG expression and assessed the corresponding expression profile changes. Whereas all MPC isolates tested could stably express EWS-FLI-1, only some sustained stable EWS-ERG expression and none could express FUS-ERG for more than 3–5 days. Only 14% and 4% of the total number of genes that were respectively induced and repressed in MPCs by the three fusion proteins were shared. However, all three fusion proteins, but neither FLI-1 nor ERG-1 alone, activated the *IGF1* promoter and induced *IGF1* expression.

**Conclusion/Significance:**

Whereas expression of different ESFT-associated fusion proteins may require distinct cellular microenvironments and induce transcriptome changes of limited similarity, *IGF1* induction may provide one common mechanism for their implication in ESFT pathogenesis.

## Introduction

Ewing's sarcoma family tumors are associated with non-random chromosomal translocations that generate fusion genes which encode aberrant transcription factors [1], [2]. The most common chromosomal translocation, associated with 85% of ESFTs is t(22;11)(q24;q12 ), which generates the EWS-FLI-1 fusion [3]. The t(21;22)(q22;q12) translocation is associated with about 10% of tumors and leads to EWS-ERG formation, whereas the remaining 1–5% of ESFT bear translocations that generate FUS-ERG, EWS-FEV, EWS-ETV1 and EWS-ETV4 fusions [1], [4]. In all cases, the transactivation domain of one of the TET (TLS/FUS, EWS, TAFII68) family of proteins is fused to the DNA binding domain-containing region of an ets transcription factor family member [1], [4].

The fusion proteins associated with ESFT are believed to provide the oncogenic stimulus that transforms primary cells, at least in part by altering their transcriptome. In NIH 3T3 cells, different ESFT-associated fusion genes have been observed to induce a similar tumor phenotype [5]. However, EWS-FLI-1 induces an oncogenic stress-type response in primary human and mouse fibroblasts [6], [7], suggesting that a distinctly permissive cellular environment may be required for EWS-FLI-1-mediated oncogenesis. Recent evidence indicates that primary bone marrow-derived MPCs are permissive for EWS-FLI-1 expression as well as its transforming effects and that EWS-FLI-1 expression may constitute the initiating event in ESFT pathogenesis [8]. In contrast to other primary or transformed heterologous cells transduced with EWS-FLI-1, MPCs expressing EWS-FLI-1 (MPC*^EWS-FLI-1^*) displayed a transcriptome consistent with survival, proliferation and invasion [8]. Among potentially relevant genes found to be induced in MPCs but not in other primary cells infected with EWS-FLI-1-containing vectors was *IGF-1,* which constitutes a key growth factor for ESFT survival [8]. These observations suggest that *IGF-1* may be an EWS-FLI-1 target in a permissive cellular context that may constitute an origin of ESFT.

Most studies on ESFT fusion proteins have been conducted on EWS-FLI-1, given that the frequency of its association with ESFT is by far the highest. Several of these studies have shown that the target gene repertoire of EWS-FLI-1 varies according to the host cell type [9], [10]. To determine whether EWS-FLI-1 and other ESFT-associated fusion proteins trigger similar responses in cells from which ESFT are believed to originate, we stably introduced *EWS-FLI1, EWS-ERG* and *FUS-ERG* into MPC and addressed the corresponding transcription profile changes. We compared these changes to those induced by *FLI1* and *ERG1* alone as well as to those induced by an isoform of *FUS-ERG* associated with acute myeloid leukemia (AML) but not ESFT [11]. Our results show that MPCs display differential permissiveness for EWS-FLI-1, EWS-ERG and FUS-ERG and that among the gene expression changes induced by the three fusion proteins only a limited fraction are shared. One of the genes observed to be induced by all three fusion proteins was *IGF1.* In the present work we provide evidence that *IGF1* is a direct target gene of ESFT fusion proteins.

## Results

### Expression of ESFT fusion proteins in mesenchymal progenitor cells

**Figure 1:**
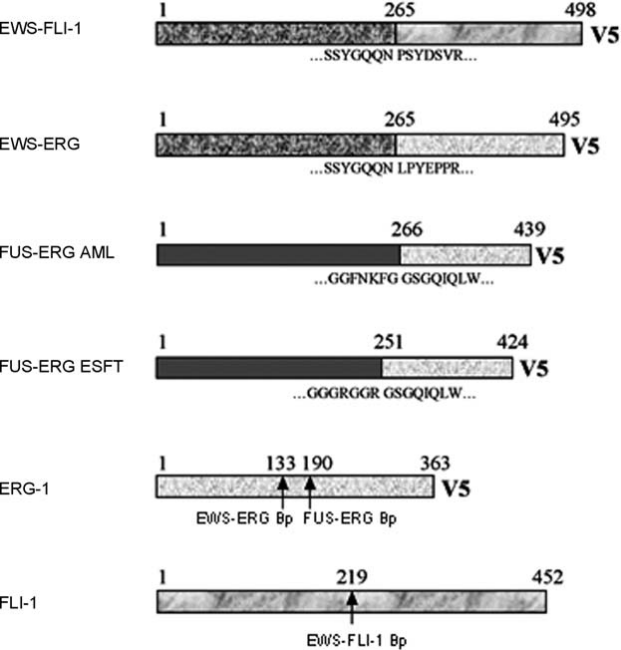
Schematic representation of EWS-FLI-1, EWS-ERG, FUS-ERG (AML) and FUS-ERG (ESFT) fusion proteins and human ERG-1 and FLI-1 proteins. Amino acid sequences at the breackpoints of each chimera are annotated and breakpoints on ERG-1 and FLI-1 are indicated by arrows.

The Ewing's sarcoma *EWS-FLI1, EWS-ERG* and *FUS-ERG* fusion genes, the AML *FUS-ERG* fusion and the human *FLI1* and *ERG1* genes were RT-PCR amplified as described in materials and methods. A schematic representation of all the constructs is shown in Figure 1. An *EWS-FLI1* mutant encoding the R340N mutation in the DNA binding domain of FLI-1 was generated to serve as a control for DNA binding-dependent versus DNA binding-independent effects. The R340N mutant (DNA-binding mutant, DBDM) has been shown to conserve some degree of transforming capacity in NIH3T3 fibroblasts despite lacking DNA binding activity [12].

**Figure 2:**
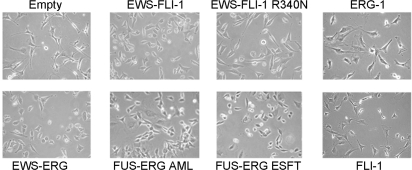
Phenotype of mouse MPCs infected with EWS-FLI-1, EWS-FLI-1 R340N mutant, EWS-ERG, FUS-ERG ESFT, FUS-ERG AML, ERG-1, FLI-1 or an empty pMSCV puro vector. Images were taken 3 days after infection (200X magnification).

Three independently isolated C57Bl6 mouse MPC populations were derived from the bone marrow, and three to four replicate infections were performed for each construct. Thus, the same MPC populations were used to test the expression and effect of each fusion and wt protein, eliminating the possibility that any difference in the effects of the proteins might reflect differences among the primary MPC batches. Expression of all three fusion proteins induced mild but readily detectable morphological changes of the MPCs, characterized by loss of the elongated spindle shape and rounding. By contrast, cells expressing the DNA binding mutant of EWS-FLI-1 maintained the characteristic MPC spindle shape as did cells expressing FLI-1 and ERG-1 alone (Figure 2). No change in the expression of mesenchymal stem cell markers, as assessed by FACS analysis, was observed in any of these populations (data not shown).

**Figure 3:**
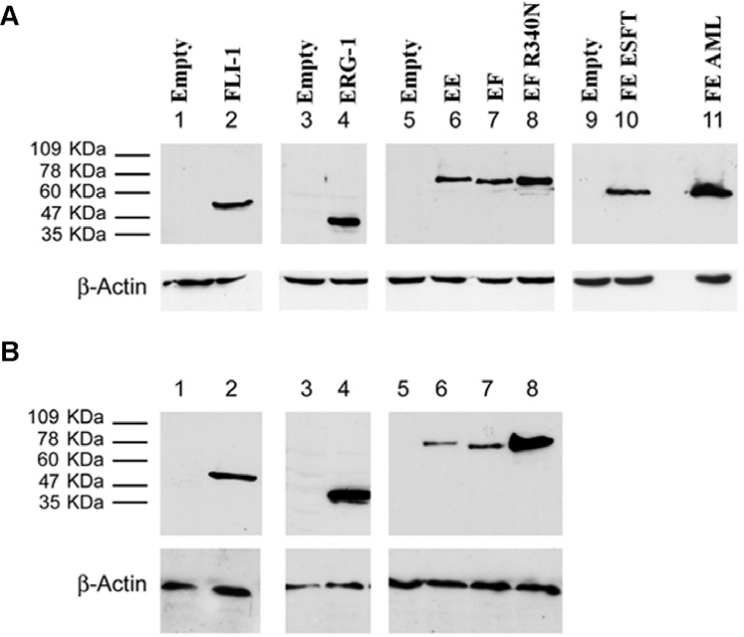
Expression in mouse MPCs of human FLI-1 and ERG-1 , ESFT-associated fusion proteins EWS-FLI-1 (EF), EWS-ERG (EE) and FUS-ERG (FE), the AML-associated FUS-ERG fusion protein variant and the EWS-FLI-1 R340N DBD mutant. Mouse MPCs were infected with human ERG-1 (A and B lane 4), human FLI-1 (A and B, lane 2), EWS-FLI-1 (A and B, lanes 7), EWS-FLI-1 R340N DBD mutant (A and B, lanes 8), EWS-ERG (A and B, lanes 6), FUS-ERG ESFT (lane 10), FUS-ERG AML (lane 11), or an empty pMSCV puro vector (A, lanes 1,3,5,9; B lanes 1, 3, 5). Cells were harvested 36 hours after the infection (A) or selected in 1.5 µg/ml puromycin for a period of 14 days (B). Protein expression was assessed by western blot analysis using a mouse anti v5 epitope (A, lanes 3–11; B, lanes 3–8) or a mouse anti human FLI-1 monoclonal antibody (A and B lanes 1–2). The secondary antibody was an HRP-conjugated goat anti mouse IgG. Monoclonal mouse anti-actin antibody was used as a loading control. With the exception of EWS-ERG in selected cells (B, lane 6), bands are representative of expression levels obtained in 3 independent MPC populations.

**Figure 4:**
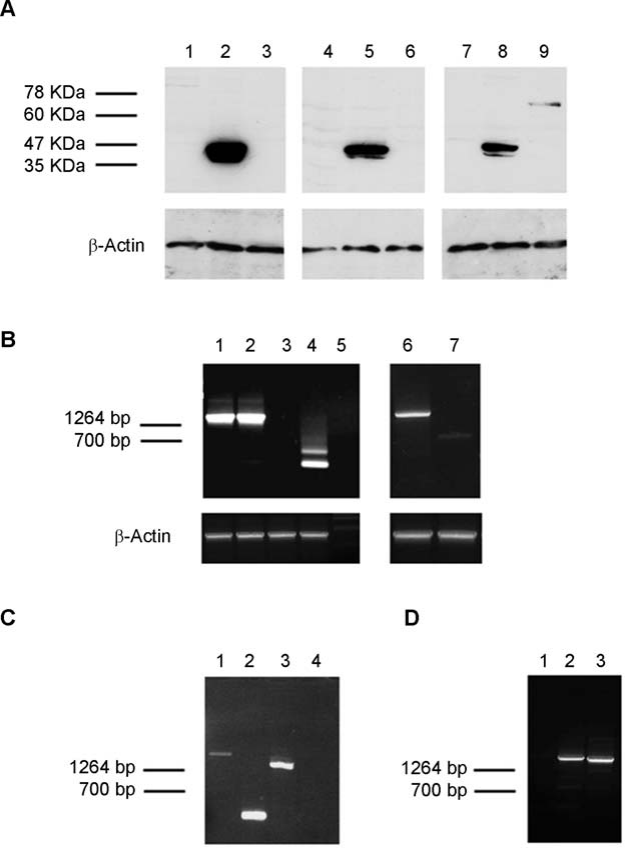
Variability of EWS-ERG expression in mouse MPCs. A) Western blot analysis: three independent MPCs populations were infected with human ERG-1 (lanes 2, 5 and 8), EWS-ERG (lanes 3, 6 and 9) or an empty pMSCV puro vector (lanes 1,4 and 7). After confirming ERG-1 and EWS-ERG expression at the protein level 36 hours following infection (data not shown), cells were selected with 1.5 µg/ml puromycin for 14 days, and protein expression was re-assessed using a mouse anti v5 epitope mAb and a goat anti mouse-HRP conjugated antibody. Monoclonal mouse anti-actin was used as a loading control. (B) RT-PCR analysis of EWS-ERG infected MPC populations. RNA was extracted from puromycin-selected MPCs populations expressing (lanes 1 and 6) or not (lanes 2 and 4) EWS-ERG protein and from puromycin-selected MPC populations infected with an empty pMSCV puro vector (lanes 3 and 7). RT-PCR was performed using primers complementary to the 5′end of EWS and at the 3′end of ERG. A control lacking RT was included (lane 5) as well as a mouse β-actin amplification RNA control. (C) Genomic DNA analysis of EWS-ERG infected MPC populations. Lanes1 and 3: puromycin-selected MPC populations lacking EWS/ ERG protein (A lane 3) and showing degraded EWS-ERG RNA (B lane 4). Lanes 2 and 4: puromycin-selected MPC populations infected with an empty pMSCV puro vector. PCR was performed using primers either at the 5′end of EWS and at the 3′end of ERG-1 (lanes 3 and 4) or primers located on the pMSCV vector (lanes 1 and 2). D) Genomic DNA analysis of FUS-ERG infected MPCs populations. Genomic DNA was extracted from puromycin-selected MPCs populations infected with ESFT-associated FUS-ERG (lane 3), AML-associated FUS-ERG (lane 2) or an empty pMSCV puro vector (lane 1). PCR was performed using primers complementary to the 5′end of EWS and to the 3′end of FUS.

Expression of the fusion and wild type proteins was tested by Western blot analysis. All of the fusion proteins as well as FLI-1 and ERG-1 were readily detected 36 hours following infection (Figure 3A). Densitometric analysis revealed that EWS-FLI-1, EWS-ERG and the ESFT-associated FUS-ERG isoform were expressed at comparable levels, whereas the DBDM, FLI-1, ERG-1 and the AML-associated FUS-ERG isoform displayed a 1.5 to 2.5 fold higher expression level (Figure S1). Expression of wild type FLI-1 and ERG-1, as well as that of the DBDM was stably maintained by MPCs infected with the corresponding viruses (Figure 3 B); EWS-FLI-1 protein expression was also stably maintained by MPCs, but at a 4-, 2- and 7 fold lower level than the DBDM and the FLI-1 and ERG-1 proteins, respectively, as assessed by densitometric analysis (Figure 3 B and Figure S1). Comparable expression levels of each of these proteins were observed in independently infected cells derived from the same primary population and in different MPC populations (data not shown). However, the same MPC populations displayed variable ability to maintain EWS-ERG expression and were unable to stably express either of the two FUS-ERG fusion proteins (Figure 4 and data not shown). The EWS-ERG fusion protein was maintained beyond 14 days of culture and selection in only one out of three MPC populations tested (figure 4A, lanes 3, 6, 9). Both genomic DNA and total RNA from cells that had lost EWS-ERG protein expression tested positive for the presence of EWS-ERG by PCR or RT-PCR using EWS-ERG specific primers. In some samples, RT-PCR analysis showed that loss of protein expression was accompanied by non-random EWS-ERG RNA processing (figure 4B, lane 4), reflected by the presence of two distinct transcripts of about 250 and 500 bp. PCR-amplification using primers complementary to the 5′end of *EWS* and the 3′end of *ERG* and subsequent sequence analysis of the amplicons revealed that the lower band was composed of the initial 5′ 175 bases of *EWS* fused to the 3′ 57 bases of *ERG* and the V5 tag. The higher band consisted of the 5′ 333 coding bases of *EWS* fused to the 3′ 139 coding bases of *ERG* and the V5 tag. In both cases the Ewing's sarcoma breakpoint was lost and translation showed no open reading frame (data not shown). No deamination was observed within the sequence suggesting the absence of RNA editing, and no point mutations were found in the EWS/ERG sequence.

Expression of both FUS-ERG fusion proteins was limited to no more than 3–5 days. Cells surviving puromycin selection tested positive for FUS-ERG DNA integration into the host genome (figure 4D) but were negative for FUS-ERG RNA expression.

Cell cycle analysis (Figure S2) showed that 36 hours following infection, prior to puromycin selection, only a small fraction of cells underwent apoptosis, with no significant difference among the different fusion gene-containining vector-infected cells (data not shown). At later time points, under puromycin selection, all of the populations displayed higher fractions of apoptotic cells. However, there was little difference in the apoptotic cell fractions among populations expressing the different fusion genes. In fact, the highest fraction of apoptotic cells seemed to be found in the EWS-FLI-1 expressing cell population, suggesting that apoptosis was not the main cause of the loss of EWS-ERG and FUS-ERG protein expression.

### Gene expression profiles of MPCs containing ESFT fusion proteins, FLI-1 and ERG-1 display limited similarity

**Figure 5:**
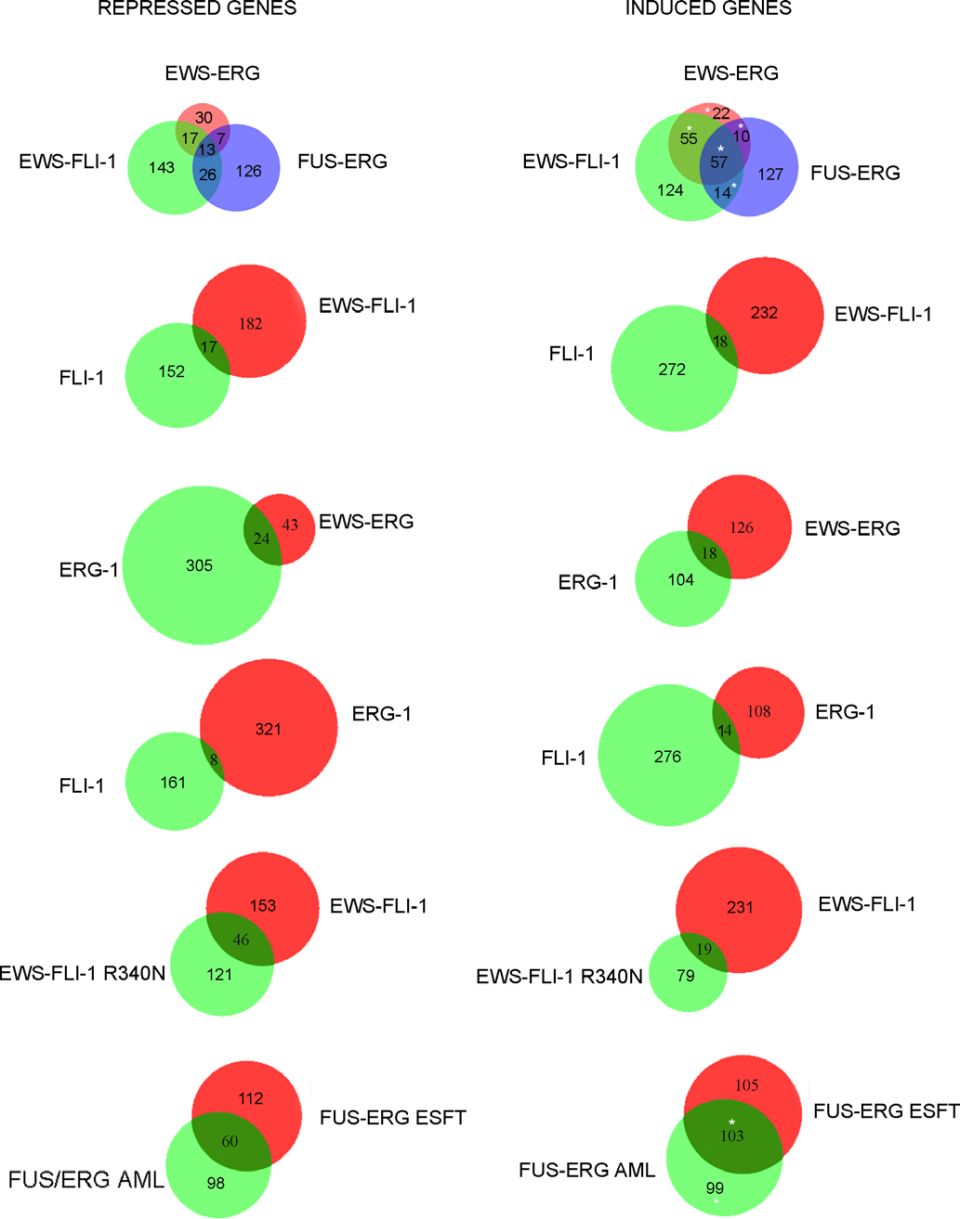
Venn diagrams indicating the extent of overlap of the transcription profiles of MPCs expressing the three ESFT- associated fusion proteins, ERG-1, FLI-1, the EWS-FLI-1 R340N mutant and the AML-associated FUS-ERG fusion protein. A white asterisk marks the areas where Igf1 is commonly present.

Transcriptional changes were analysed 36 hours and 4 days following infection, when expression of all of the fusion proteins and that of the corresponding wild type ets family proteins was comparable. No significant difference in the gene expression profile induced by the various fusion genes at 36 hours and 4 days was observed (data not shown). The number of genes observed to be induced and repressed differed among cells expressing the three fusion proteins, with a higher number of genes being affected by EWS-FLI-1 and FUS-ERG than by EWS-ERG. Using a 5% false discovery rate, cells expressing EWS-ERG displayed 67 repressed and 144 induced genes, whereas EWS-FLI-1 and FUS-ERG expressing cells had 199 and 172 repressed and 250 and 208 induced transcripts, respectively (Table S1, Figure 5).

Consistent with the notion that a predominantly gene expression inducing transcriptional activity is associated with ESFT fusions [13], [14], pairwise comparison revealed greater similarity among the induced than among the repressed transcript repertoires (Figure 5). Thus, of all of the genes induced in cells infected with EWS-FLI-1 and EWS-ERG retroviruses, ∼40% were shared whereas only ∼13% of those that were repressed were common. EWS-ERG and FUS-ERG expressing cells shared about 21% of the induced but less than 12% of repressed transcript repertoire, similar to EWS-FLI-1 and FUS-ERG expressing cells that shared 22% of their induced and 10% of their repressed genes (Table S1 and Figure 5). Fifty-seven induced (14% of the total) and 13 repressed (4% of the total) transcripts were common to all three fusion proteins (Figure 5 and Table S1). The same comparison revealed somewhat greater similarity between EWS-FLI-1 and EWS-ERG induced genes than between genes induced by either fusion and those induced by FUS-ERG, suggesting a potentially important role of the N-terminal portion of the fusions in target gene selection. In contrast to the limited but nevertheless significant overlap of induced and repressed transcripts in response to the three fusion proteins, ERG-1 and FLI-1 shared very few common targets in MPCs. Whereas ERG-1 induced 122 and repressed 329 transcripts, and FLI-1 induced 290 and repressed 169 transcripts, only 14 of the induced (3.5%) and 8 of the repressed transcripts (1.6%) were common to the two transcription factors (Figure 5).

Similarity between the effects of FLI-1 and ERG-1 and those of their corresponding fusion proteins was also limited to a restricted number of genes (Figure 5). Thus, about 20% of all of the genes induced and repressed by EWS-ERG were also induced and repressed by ERG-1 alone and less than 8% of genes induced and repressed by EWS-FLI-1 were induced and repressed by FLI-1 alone.

The role of the DNA binding domain in the transcriptional activity of EWS-FLI-1 fusion protein was addressed by comparing the transcriptional activity of wt EWS-FLI-1 with that of the DBDM. The EWS-FLI-1 DBDM induced 98 and repressed 167 transcripts in MPCs compared to, respectively, 250 induced and 199 repressed transcripts by wt EWS-FLI-1, suggesting that impairment of EWS-FLI-1 DNA binding affects the transcriptional activation capacity of the fusion more strongly than its transcriptional repression capacity. Pairwise comparison revealed that only 65 out of a total of 649 (∼10%) genes that were induced and repressed were shared (Figure 5).

Finally, comparison between the effects on MPCs of FUS-ERG fusions found in ESFT and AML revealed a similar number of induced and repressed transcripts. Of the total induced transcripts by the two fusion proteins, about 34% were common whereas about 22% of all of the repressed transcripts were shared between cells expressing the two fusions (Figure 5, Table S1).

### Distinct and common candidate target genes of ESFT fusion proteins and their component ets family members

Expression of fifteen of the genes that were found to be significantly up- or down-regulated in MPC expressing EWS-FLI1, EWS-ERG and FUS-ERG was validated by quantitative real-time PCR in the corresponding cells as well as in FLI-1 and ERG-1 expressing counterparts (a complete list of up- and downregulated genes is provided in Table S2, whereas shared genes are listed in Table S3). *DHH, PODXL*, *KRT119* and *CDH5* were up-regulated in FLI-1 and ERG-1-expressing cells as well as in all three fusion protein expressing MPCs (Table 1). By contrast, *CITED1, SFRP4, IGF1, IGFBP5, IGFBP3* and *ENO3* were among genes that were upregulated in all three fusion protein containing cells but not in cells expressing FLI-1 and ERG-1 alone (Table 1). Several other genes, including *DCN, MT2, DKK3, DKK2* and *MMP3* displayed movement in one or two fusion protein containing cells but not in FLI-1 and ERG-1 retrovirus-infected cells.

**Table 1:** Real time PCR validation of ESFT fusion protein- and wild type FLI-1 and ERG-1-induced and repressed genes 36 hours following infection

Gene symbol	EWS-ERG		EWS-FLI-1		FUS-ERG		ERG-1		FLI-1	
	Fold increase/decrease	SD	Fold increase/decrease	SD	Fold increase/decrease	SD	Fold increase/decrease	SD	Fold increase/decrease	SD
CITED-1	49.91	0.16	461.61	0.1	80.21	0.08	<I 2 I		<I 2 I	
*DHH*	28.24	0.66	28.61	0.19	22.84	0.49	3.1	0.51	14.16	0.51
*SFRP4*	79.21	0.37	57.76	0.56	42.84	0.21	<I 2 I		2.12	0.05
*IGF-1*	5.31	0.125	8.76	0.2	2.26	0.08	<I 2 I		<I 2 I	
*IGFBP5*	25.23	0.135	47.8	0.14	11.72	0.105	<I 2 I		<I 2 I	
*GFBP3*	4.98	0.3	2.34	0.3	2.83	0.27	<I 2 I		<I 2 I	
*PODXL*	4.84	0.15	10.72	0.13	3.98	0.1	8.16	0.37	30.26	0.28
*KRT1-19*	33.46	0.44	152.6	0.41	18	0.07	2.04	0.76	9.54	1
*CDH5*	26.92	1.64	24.9	0.46	106.41	0.45	34.27	0.5	251.17	0.3
*ENO3*	5.17	0.18	8.3	0.45	2.49	0.06	<I 2 I		<I 2 I	
*DCN*	−5.3	0.15	−10	0.18	<I 2 I		<I 2 I		<I 2 I	
*MT2*	<I 2 I		<I 2 I		2.39	0.26	<I 2 I		<I 2 I	
*DKK3*	−2.5	0.7	<I 2 I		<I 2 I		<I 2 I		<I 2 I	
*DKK2*	<I 2 I		−3.3	0.2	<I 2 I		<I 2 I		<I 2 I	
*MMP3*	<I 2 I		<I 2 I		6.95	0.09	<I 2 I		<I 2 I	

The indicated fold increase or decrease (−) and the corresponding standard deviation are representative of three independent experiments. Lower than two-fold increases or decreases in absolute value are indicated.as <I 2 I

Comparison of the expression of these same genes in cells expressing EWS-FLI-1 and the EWS-FLI-1 DBDM revealed that *IGFBP3, PODXL, KRT119* and *CDH5* were induced in both cell types, albeit substantially more strongly in wt EWS-FLI-1 expressors for all except *IGFBP3* (Table 2). By contrast, *DHH, IGF1, IGFBP5* and *ENO3* were upregulated in wt EWS-FLI-1 expressing cells only. *CITED1* was upregulated in both but more than 150 times more strongly in EWS-FLI-1 expressing cells (Table 2).

**Table 2:** Real time PCR validation of EWS-FLI-1- and EWS-FLI-1 R340N-induced and repressed genes 36 hours following infection

Gene symbol	EWS-FLI-1		EWS-FLI-1 R340N	
	Fold increase/decrease	SD	Fold increase/decrease	SD
CITED-1	461.61	0.1	3.19	0.17
*DHH*	28.61	0.19	<I 2 I	
*SFRP4*	57.76	0.56	<I 2 I	
*IGF-1*	8.76	0.2	<I 2 I	
*IGFBP5*	47.8	0.14	<I 2 I	
*IGFBP3*	2.34	0.3	10.16	0.13
*PODXL*	10.72	0.13	4.41	0.18
*KRT1-19*	103.15	0.41	18.9	0.36
*CDH5*	24.9	0.46	2.48	1.14
*ENO3*	8.3	0.45	<I 2 I	
*DCN*	−7.6	0.18	−3.3	0.23
*MT2*	<I 2 I		−2.7	0.12
*DKK3*	<I 2 I		<I 2 I	
*DKK2*	−3.3	0.198	<I 2 I	

The indicated fold increase or decrease (−) and the corresponding standard deviation are representative of three independent experiments. Lower than two-fold increases or decreases in absolute value are indicated.as <I 2 I

When the effects of the two FUS-ERG fusions in MPCs were compared, all of the genes tested displayed movement in the same direction. The only difference was that the degree of induction of each gene was 1.25 (*DHH*) to more than 10 (*IGFBP3*) times greater in cells expressing the AML-associated fusion (Table 3). It is conceivable that the stronger induction associated with the AML than with the ESFT fusion is a reflection of the higher expression level of the AML fusion protein that appears to be tolerated in MPCs.

**Table 3:** Real time PCR validation of FUS-ERG fusion protein-induced and repressed genes 36 hours following infection

Gene symbol	FUS-ERG AML		FUS-ERG ESFT	
	Fold increase/decrease	SD	Fold increase/decrease	SD
CITED-1	229.28	0.03	80.2	0.08
*DHH*	28.62	0.16	22.84	0.49
*SFRP4*	108.67	0.09	42.83	0.21
*IGF-1*	5.97	0.06	2.26	0.08
*IGFBP5*	103.49	0.13	11.7	0.11
*IGFBP3*	34.1	0.53	2.83	0.27
*PODXL*	20.3	0.11	3.98	0.09
*KRT1-19*	71.13	0.146	18	0.07
*CDH5*	544.82	0.54	106.41	0.45
*ENO3*	5.19	0.08	2.49	0.06
*MMP3*	16.93	0.13	6.95	0.09
*MT2*	6.3	0.11	2.39	0.26
*DKK3*	−2.5	0.05	<I 2 I	
*DKK2*	−2	0.11	<I 2 I	

The indicated fold increase or decrease (−) and the corresponding standard deviation are representative of three independent experiments. Lower than two-fold increases or decreases in absolute value are indicated as <I 2 I

Among the other putative gene targets, *CITED1*, which is a major discriminator of human ESFT with respect to other sarcomas [15] was strongly induced by the fusion proteins but only weakly by the DBDM. *DHH* and *SFRP4* were both induced by wt fusion proteins but not by the DBDM, whereas *PODXL, KRT119* and *CDH5* were induced both by FLI-1 and ERG-1 alone and by the EWS-FLI-1 DBDM. These observations suggest that the DBDM conserves some transcriptional or transcription co-factor activity, consistent with the report that it maintains a degree of transforming ability in NIH3T3 cells [12].

### EWS-FLI-1 recognizes the IGF1 promoter

**Figure 6:**
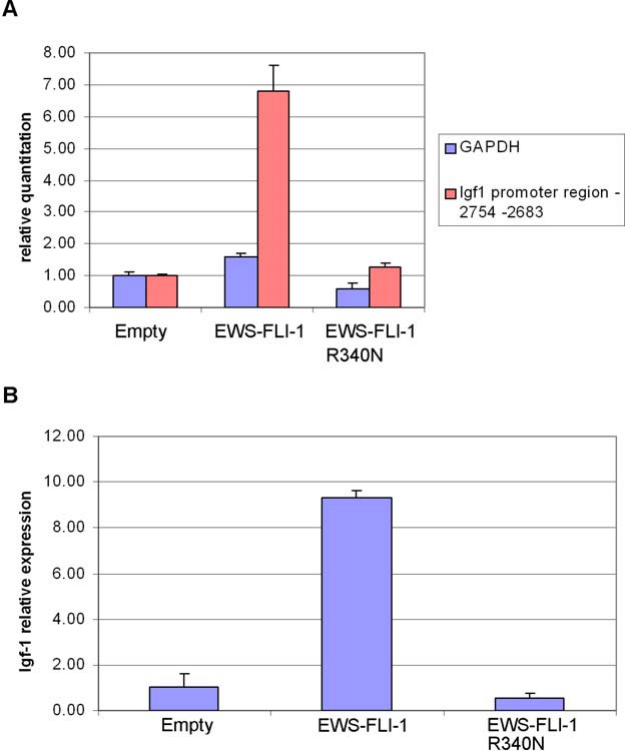
In vivo interaction of EWS-FLI-1 with the region from –2754 to –2683 of the murine IGF1 promoter. A) Quantitative RT-PCR analysis was performed on chromatin immunoprecipitates obtained from mouse MPCs expressing EWS-FLI-1, EWS-FLI-1 R340N mutant or an empty pMSCV vector. Primers specific for murine GAPDH DNA were used as input DNA control. Error bars reflect results of triplicate experiments. (B) IGF1 mRNA induction by EWS-FLI-1 and EWS-FLI-1 R340N in the MPCs used for the ChIP experiments. Quantitative RT-PCR was performed using a murine IGF1 TaqMAN probe (applied biosystems).

To address the interaction between EWS-FLI-1 and the *IGF1* promoter *in vivo*, chromatin immunoprecipitates (ChIP) from lysates of mouse MPCs expressing EWS-FLI-1, the DBDM and the empty vector were subjected to quantitative RT-PCR using primers specific for the region stretching from –2754 nucleotides to –2683 nucleotides upstream of the exon 1 start codon (ENSMUSE00000369489) of the murine *IGF1* promoter (Figure 6A). This 70 bp stretch corresponds to the sequence annotated as –408 to –338 of the murine *IGF1* promoter in a recent report by Alfieri et al. [16] and is included in the putative murine *IGF1* promoter region GXP_41004 defined by the Genomatix (http://www.genomatix.de) genome annotation tool “Eldorado”. This region shows the highest similarity (96%) to the human *IGF1* promoter region GXP_79580 and, by Genomatix analysis, is suggested to contain several potential trancription factor binding sites including ets binding sites. Relative to empty vector-expressing cells, a 5 fold increase in *IGF1* promoter occupancy by the fusion protein was observed in ChIP assays directed against the anti-V5 epitope on the EWS/FLI-1 fusion protein.

EWS-FLI-1 expressing cells displayed a 9 fold increase in *IGF1* message as measured by quantitative real time PCR (Figure 6B). No *IGF1* promoter occupancy was observed in chromatin immunoprecipitates from the DBDM expressing cells, which also displayed no induction of *IGF1* transcripts (Figure 6). Similarly, no enrichment was observed using a second set of primers amplifying a region located about 14000 bp upstream of the *IGF1* exon 1 start codon containing no putative ets binding sites (data not shown).

### Modulation of human *IGF1* promoter activity in human mesenchymal stem cells

The effect of the various fusion proteins on murine *IGF-1* promoter activity was tested in MPCs using a luciferase reporter assay, but a high background rendered interpretation of the results difficult in these cells, prompting us to use human mesenchymal stem cells (hMSC). Our recent observations have shown that hMSC infected with an *EWS-FLI1* containing retrovirus can sustain EWS-FLI-1 expression and display a 20 fold induction of *IGF1* [17]. Studies by others have shown that downregulation of EWS-FLI-1 expression in ESFT cell lines results in the emergence of an hMSC phenotype [18], supporting the notion that hMSCs may provide an ESFT origin. We therefore used hMSCs to further explore the relationship between ESFT fusion proteins and *IGF1*. A 1682 nucleotide (nt) region of the human *IGF1* promoter upstream of the exon 1 start codon (Ensemble ENSE00001194172) was isolated and ligated to sequences encoding the luciferase reporter gene in the pGL3 plasmid. This promoter region has been shown to be functional or to at least contain functional portions of the promoter in different *IGF1* expressing cells, including macrophages [19] and the EWS-FLI-1 expressing Ewing's sarcoma cell line SK-N MC [20]. A 6 fold increase in luciferase activity in SK-N MC cells transfected with the –1682pGL3 construct was observed compared to that in cells bearing a promoterless construct (data not shown). *IGF1* promoter analysis using the Genomatix (http://www.genomatix.de) genome annotation tool “Eldorado” showed that the region from –1682 to +1 contains 2 separate putative promoters upstream of the exon I start codon: GXP79580 from –782 to –64 and GXP641910 from –1664 to –962.

**Figure 7:**
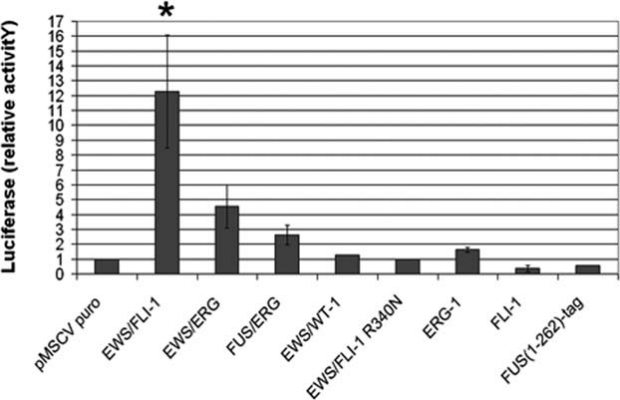
IGF1 promoter activity in human mesenchymal stem cells expressing the three ESFT-associated chimeric proteins EWS-FLI-1, EWS-ERG and FUS-ERG. Promoter activity in the presence of the EWS-FLI-1 R340N mutant, the FLI-1 and ERG-1 wild type proteins, the EWS-WT1 fusion and the FUS(1-262)-tag protein are also shown. Luciferase activity is reported as a value relative to that measured in vector transfected cells; error bars represent the S.E.M. of three independent experiments. The significance (p<0.01) of luciferase induction is indicated by an asterisk.

MSCs were co-transfected with the –1682pGL3 construct together with either an empty pMSCV or a pMSCV construct containing sequences encoding the EWS-FLI-1, EWS-ERG or FUS-ERG fusion. The EWS-WT1 fusion associated with desmoplastic small round cell tumors (DSRCT,[21]) and a truncated protein composed of the first 262 amino acids of FUS with a short tag were used as controls along with FLI-1 and ERG-1 proteins. A 12-fold increase in luciferase activity was observed in EWS-FLI-1 expressing cells compared to empty pMSCV transfected cells, whereas a 4.5- and a 2.5-fold increase were observed, respectively, in cells expressing EWS-ERG and FUS-ERG (Figure 7). The capacity of the three fusions to mediate *IGF1* promoter activation in human MSCs reflected their respective ability to induce *IGF1* expression in mouse mesenchymal progenitor cells, as assessed by real time PCR (Table 1). By contrast, luciferase activity in cells expressing EWS-WT1, the FUS (1-262) control protein, FLI-1 or ERG-1 was comparable to that of cells transfected with the empty vector suggesting that neither of the two ets family members nor the EWS and FUS portions alone were able to activate the *IGF1* promoter. Consistent with our initial observations, the DBDM did not induce any increase in luciferase activity (Figure 7). The integrity of the DNA binding domain of FLI-1 in the context of the fusion is therefore necessary for EWS-FLI-1 mediated activation of the *IGF1* promoter in MPCs and hMSC (Table 2).

**Figure 8:**
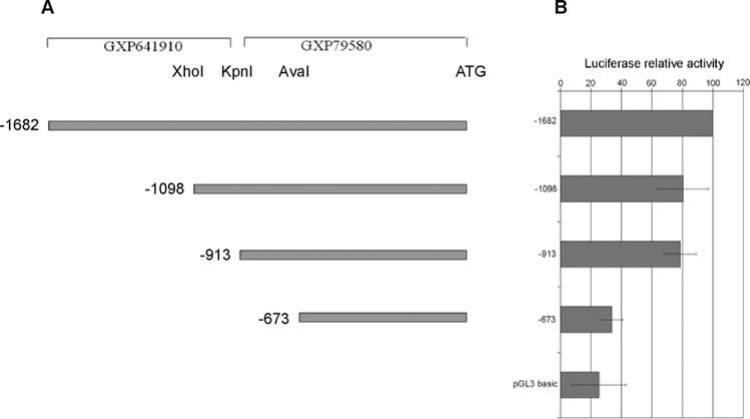
Schematic representation of *IGF1* promoter fragments cloned into the pGL3 basic vector (A) and the corresponding luciferase activity (B) in human mesenchymal stem cells expressing EWS-FLI-1. Restriction sites used for cloning are indicated. The exon 1 start codon, putative promoter regions GXP79580 and GXP641910 according to GEM analysis (Genomatix) are shown. Levels of luciferase activity, calculated as in Figure 6, are normalized to values obtained with the –1682 construct which was arbitrarily set to 100%. Error bars represent the S.E.M. of three independent experiments.

GEMS analysis [22], used to search for transcription factor binding sites (MatInspector) and sequence models (ModelInspector), of the –1682 to +1 region of the human *IGF-1* promoter, revealed the presence of potential binding sites for several different transcription factors, including ets family members, supporting the possibility of direct interaction of Ewing's sarcoma fusion proteins with the *IGF-1* promoter. Comparative Genomix analysis, on the other hand, showed no conserved putative ets binding sites in this region, whereas other transcription factor binding sites, such as RFX1, were found to be conserved. To define the relevance of the 2 putative promoter regions GXP79580 and GXP641910, identified by Genomatix analysis, 3 additional luciferase fusion plasmids were generated by restriction enzyme cleavage and ligation to the pGL3 vector (Figure 8 A). Constructs –1098pGL3 and –913pGL3, generated respectively by XhoI/HindIII, and KpnI/HindIII digestion, included the entire GXP79580 promoter, a short sequence of GXP641910 being included in the –1098pGL3 construct. The –673pGL3 plasmid, obtained by AvaI/HindIII digestion, contained only part of the GXP79580 promoter.

Luciferase activity, measured in human mesenchymal stem cells transfected with an EWS-FLI-1 containing plasmid together with the –1098pGL3 or the –913pGL3 construct, was similar, a 20% reduction of activity being observed in both cases compared to cells into which the entire promoter region –1682 had been introduced. A more robust reduction of activity (70%) was observed when the –673pGL3 construct was introduced.

Taken together these data suggest that both GXP79580 and GXP641910 regions contribute to EWS-FLI-1 mediated *IGF1* promoter activity, although a more substantial contribution is made by the GXP79580 region. The observed difference in contribution could be explained by the loss of potential binding sites for different transcription factors within this region. Although ets binding sites are plausible candidates for EWS/FLI-1 mediated *IGF-1* promoter activation, other sequences relevant to *IGF-1* promoter activity could be present in the deleted segments. Among many other binding sites lost in the –673 construct were an E-box, a STAT binding site which forms a sequence model (Genomatix modelInspector) together with an ets binding site, and a Myc associated zinc finger binding site (MAZF). Mutagenesis experiments to define the role of different potential ets and other transcription factor binding sites in the regulation of IGF-1 expression are ongoing.

## Discussion

The distinguishing feature of Ewing's sarcoma is the expression of an aberrant transcription factor encoded by a fusion gene resulting from a non-random chromosomal translocation. In all cases the fusion protein is composed of the amino terminal portion of a TET family member that provides a potent transactivating domain and the DNA binding domain of one of several possible ets family members. In more than 99% of cases, the TET family member is EWS and in 85–90% of cases the ets family member is FLI-1. EWS is fused to ERG in 5–10% of cases whereas FUS-ERG is found in less than 1% of cases [1], [2], [4], [12]. The difference in frequency of association of the fusion proteins with ESFT is currently unexplained, and could conceivably reflect the relative frequency of the corresponding chromosomal breaks. However, our present observations using mouse MPCs suggest that primary mesenchymal stem cells display a markedly different degree of permissiveness for the three fusion proteins. Whereas expression of EWS-FLI-1 was tolerated in all of the cell batches tested, expression of EWS-ERG was restricted to a fraction of the batches while stable expression of FUS-ERG could not be achieved. The observed differential permissiveness correlates with the relative frequency at which each fusion accompanies ESFT cases, suggesting distinct windows of opportunity for the different fusion proteins to display their putative transforming properties in MPCs.

Mechanisms whereby MPCs restrict expression of EWS-ERG and FUS-ERG remain to be elucidated. Whereas in some cases the specific RNA was not detectable or appeared degraded, in others protein expression could not be detected despite maintenance of transcripts of appropriate length. It is conceivable that discrete stages of MPC differentiation may account for the observed differences in permissiveness. Alternatively, MPCs may be composed of functionally heterogeneous cell subsets that cannot be distinguished on the basis of the restricted number of phenotypic markers used to characterize them. A plausible scenario may be that a majority of these putative subsets display a milieu that is favourable for EWS-FLI-1 expression and function, whereas only rare subsets may tolerate expression of FUS-ERG. Thus, the composition or differentiation stage of MPC populations may determine whether or not expression of ESFT-associated fusion proteins other than EWS-FLI-1 may be sustained.

The observed difference in permissiveness for fusion protein expression could not be attributed to functional differences among MPC batches because the same batches were used for expression of all of the contructs.

Although EWS-FLI-1 has been shown to have the capacity to transform primary MPCs that express functional p53 and p19^ARF^ [8], EWS-FLI-1 expression and transforming potential in primary unsorted bone marrow cells were found to be facilitated by inactivating *TP53* mutations [23]. Alterations in signaling pathways relevant to oncogenesis may therefore provide at least one mechanism that renders primary cells permissive for ESFT-associated fusion protein expression and susceptible to the corresponding transforming properties.

Interestingly, FLI-1 and ERG-1 displayed higher potential target gene similarity in the context of EWS-associated fusion proteins than alone. It is possible that several common FLI-1 and ERG-1 candidate targets are undetected because of the weakness of the intrinsic transactivation domain of the two transcription factors. The potency of EWS transactivation may provide a mechanism to unmask such putative targets. The ability of EWS to interact with numerous proteins implicated in transcriptional regulation and RNA processing [24], [25], [26], [27], [28], [29] may supply an alternative explanation. Thus, it is possible that a combination of EWS and its binding partners confer upon FLI-1 and ERG-1 the ability to induce expression of common genes. Of the fraction of induced and repressed transcripts that were common to the three fusion proteins, most are not known to be directly implicated in transformation, and it is possible that the three fusion proteins use distinct mechanisms to transform primary cells, resulting nevertheless in tumors with an indistinguishable phenotype as assessed by conventional histology and immunohistochemistry. One potentially important functional similarity among the fusion proteins in the context of ESFT pathogenesis, however, is their ability to induce *IGF1.*

Recently, EWS-FLI1 was shown to transform MPCs and produce tumors with an ESFT-like phenotype in immunodeficient mice [8]. Three potentially relevant genes that were found to be strongly induced were *IGF1, IGFBP3* and *IGFBP5*. IGF-1 is believed to play a critical role in ESFT growth and development [8], [30], [31], [32]. Transformation of NIH3T3 fibroblasts by EWS-FLI-1 requires expression of IGF-1R [33] and blockade of the receptor using antibodies [31] or small molecule receptor tyrosine kinase (RTK) inhibitors [8], [32] strongly inhibits ESFT cell growth. The present results indicate that *IGF1, IGFBP3* and *IGFBP5* are induced by all of the fusion proteins tested but not by FLI-1 and ERG-1 alone. Importantly, induction of *IGF1* and *IGFBP5* requires integrity of the DBD of FLI-1 within the fusion protein as demonstrated by the inability of the DBDM to induce either molecule. Quantitative RT-PCR results from chromatin immunoprecipitates argue that EWS-FLI-1 interaction with the *IGF1* promoter occurs *in vivo*. The observations that EWS-FLI-1 activates the *IGF1* promoter as assessed by luciferase reporter assays in human MSCs provide further support to the notion that *IGF1* is a target of EWS-FLI-1.

By contrast, the DBDM could induce *IGFBP3* suggesting that in MPCs EWS-FLI-1 does not require integrity of the DBD for *IGFBP3* upregulation. The observation that *IGFBP3* was induced in a non-DBD-dependent manner by EWS-FLI-1 in MPCs in apparent contrast to the recent report that *IGFBP3* is a direct target of EWS-FLI-1 which is down regulated as a result of EWS-FLI-1 expression in an ESFT cell line [34]. These contrasting results may be explained, in part, by cell state differences. ESFT cell lines reflect late stage tumor progression where the effect of EWS-FLI-1 may be modulated by a variety tumor stage-associated factors. The effect of EWS-FLI-1 and the other fusion proteins on MPCs, on the other hand, was evaluated in primary cells at an early time point following expression, prior to transformation and tumor development.

Growth hormone induces IGF-1 and the robust growth hormone spurt during puberty may help create favorable conditions for the initial development of ESFT from mesenchymal stem cells harboring the appropriate chromosomal translocations and expressing the corresponding fusion proteins. Subsequent maintenance of tumor growth may require tumor cell-autonomous IGF-1 production and *IGF1* induction may provide one mechanism whereby EWS-FLI-1 and its ESFT-associated relatives ensure tumor growth and progression.

## Materials and Methods

### Mesenchymal stem cells and cell lines

Mouse mesenchymal progenitor cells (MPCs) were isolated from bone marrow of adult C57BL/6, wild type mice according to the methods described in [35], and cultured on fibronectin-coated plates (Sigma) in medium containing 2% dialyzed FCS (Sigma), 10 ng/ml EGF (Sigma), 10ng/ml PDGF-BB (R&D Systems) and 10ng/ml recombinant human leukemia inhibitory factor (Chemicon). Mesenchymal stem cells markers expression and differentiation assays were performed as previously described [8].

Human mesenchymal stem cells were obtained as previously described [36], [37] from femoral head bone marrow of patients undergoing total hip replacement according to the guidelines of the ethical committee protocol 01-172 and after informed consent of the patients. MSCs were cultured at low confluence in IMDM, 10% FCS, 10 ng/ml PDGF-BB (PeProtechEC, London,UK) and were tested for multilineage differentiation into adipocytes, chondrocytes and osteoblasts [36], [37]. The SK-N-MC cell line was from ATCC (Rockville, MD).

### Cell cycle analysis

DNA content and cell cycle analysis were performed by flow cytometry. Cells were centrifuged, and the pellets were gently resuspended in 1.5mL hypotonic propidium iodide solution (PI, 50 µg/mL in 0.1% sodium citrate plus 0.1% Triton X-100; Sigma). The tubes were kept at 4°C in the dark overnight. The PI-fluorescence of individual nuclei was measured by flow cytometry using standard FACScan equipment (Becton Dickinson, Mountain View, CA).

### Constructs and cDNA Cloning

A cDNA clone encoding the type 2 EWS-FLI-1 fusion protein was amplified from the SK-N-MC Ewing sarcoma cell line by RT-PCR. The amplified fragment carrying an in frame v5 tag at the 3′extremity was digested with XhoI and Hpa I and inserted into the pMSCV Puro retroviral expression vector (BD Biosciences Clontech, Palo Alto, CA).

The EWS-FLI-1 R340N DBDM was amplified by PCR using the wt sequence as template, with the following primers:

hEWS forward XhoI:

5′CCGCTCGAGCCACCATGGCGTCCACGGATTACAG 3′;

hFLI-1 reverse R340N:

5′ ATCATAGTAATAATTGAGGGCCCGGCTCAGCTTGTC 3′;

hFLI-1 forward R340N:

5′ CGACAAGCTGAGCCGGGCCCTCAATTATTACTATGA 3′;

V5 reverse Hpa I (including a stop codon):

5′ GTTAACTCACGTAGAATCGAGACCGAGGAGAGGGTTAGGGATAGGCTTACC 3′.

The amplified fragment was XhoI-HpaI-digested and inserted into the pMSCVPuro retroviral vector.

EWS-ERG and ERG-1 fragments were amplified from surgically resected Ewing's sarcoma tissue by RT-PCR.

Amplified fragments, carrying a BglII restriction site at the 5′end were inserted, using the pcDNA3.1/V5-HisTOPO TA cloning kit (Invitrogen, Carlsbad, CA), into the corresponding expression vector in frame with the V5 epitope tag. The EWS-ERG-v5 and the ERG-1-v5 fragments were digested with BglII and PmeI and inserted into the BglII/HpaI-digested pMSCV Puro vector. Human FLI-1 cDNA was amplified from hMSC RNA and inserted into EcoRI/XhoI-digested pMSCV Puro. cDNA clones encoding the human FUS-ERG AML and FUS-ERG ESFT fusions were obtained by PCR and inserted into BglII/HpaI-digested pMSCV Puro. Fragments encoding exons 1-7 or exons 1-6 of human TLS/FUS, amplified from the EST clone IMAGE:3345294, and a fragment encoding the C-terminal portion, starting at exon 9, of human ERG, amplified from the EWS-ERG-v5 clone, were used in the PCR reactions.

The primer sequences were:

External ERG-v5-reverse 5′GTTAGGGATAGGCTTACCTTCGAACCGCGGGCCGTAGTAAGTGCCCAG 3′

External BglII FUS forward

5′GGAAGATCTTCCCCACCATGGCCTCAAACGATTATACC 3′

Internal for FUS-ERG AML:

Forward5′GCTTCAATAAATTTGGTGGCAGTGGCCAGATCCAGCTTTGGCAGTTCC3′

Reverse 5′AGCTGGATCTGGCCACTGCCACCAAATTTATTGAAGCCACCAC3′

Internal for FUS-ERG ESFT:

Forward 5′GCCGTGGAGGCAGAGGCAGTGGCCAGATCCAGCTTTG 3′

Reverse 5′CAAAGCTGGATCTGGCCACTGCCTCTGCCTCCACGGCCACCT 3′

A cDNA control encoding the first 262 amino acids of the human TLS/FUS protein followed by a tail of 54 random amino acids was obtained by PCR and inserted into pMSCV Puro. The 54 random amino acid tail displayed no homology in a BLAST search and contained none of the motifs present in the ERG-1 or FLI-1 proteins as assessed by the SMART- Simple Modular Architecture Research Tool [http://smart.embl-heidelberg.de]. All constructs were sequence-verified.

### Retrovirus generation and infection

Expression of EWS-FLI-1, EWS-ERG, FUS-ERG (AML), FUS-ERG (ESFT) , ERG-1 and FLI-1 in MPCs was achieved using the Retroviral Gene Transfer and Expression (BD Biosciences Clontech), according to the manufacturer's recommendations. Infected cells were selected in 1.5 µg/ml puromycin for a maximum of 14 days.

### RNA and genomic DNA extraction and Reverse Transcription PCR

Total RNA was extracted using an RNeasy Mini Kit (Qiagen, Hilden, Germany) and genomic DNA was isolated using a DNeasy Tissue Kit (Qiagen) according to the manufacturer's recommendations. RT-PCR was performed using Super Script one step RT-PCR with the platinum Taq kit (Invitrogen, Carlsbad, CA).

### Western blot analysis

Cell lysis, SDS-PAGE and blotting were done according to standard procedures and immunodetection was performed using chemoluminescent substrate kits from Amersham according to the manufacturer's recommendations. Primary antibodies used were: monoclonal mouse anti-V5epitope antibody (Invitrogen), mouse anti human FLI-1 monoclonal antibody (G146-222 BD- Pharmingen, San Diego, CA), monoclonal mouse anti-actin (Sigma) and mouse IgG (Sigma). The goat anti-mouse HRP conjugated secondary antibody was from Amersham. Densitometric analysis was performed using the public domain image processing and analysis program NIH image J (http://rsb.info.nih.gov/ij/).

### cDNA array hybridization and cDNA array analysis

For each comparison 3 independently isolated MPC populations were used and 3 independent infections were performed. Total RNA was extracted at 36 hours following infection. The quality of RNA was verified by an Agilent RNA 600 nanoassay and by measuring the 260/280 absorbance ratio. Quality-tested total RNA was amplified using the RiboAmp RNA Amplification Kit (Arcturus, Moutainview, CA) and processed using a reverse transcription based method of label incorporation to yield labeled cDNA as previously described [8]. Following hybridization and washing, microarrays were imaged using a DNA microarray scanner (agilent technologies) and array analysis and quality control were performed as described in [8].

### Statistical analysis of the expression data

RNA derived from EWS-FLI-1, EWS-ERG, FUS-ERG (AML), FUS-ERG (ESFT), ERG-1 and FLI-1 expressing MPC populations were all subjected to microarray analysis; five m17k microarrays (among which 2 were dye swaps) were performed for each population, comparing cells expressing the protein of interest with the corresponding empty vector control cells. Differentially expressed genes were then identified with the rank product method [38] as implemented in the “RankProd” Bioconductor package [39]. We retained for further analysis the clones identified as differentially expressed with a false discovery rate of 5%. The statistical significance of the overlap between differentially expressed genes in different translocations was evaluated with Fisher's exact test.

### Real time quantitative RT-PCR

cDNA was obtained using an M-MLV reverse transcriptase and RNAse H minus (Promega). Typically 500 ng of template total RNA and 250 ng of random hexamers were used per reaction. Real time-PCR amplification was performed in an ABI Prism 7700 instrument (Applied Biosystems), using Taq Man Universal PCR mastermix and Assays-On-Demand Taq Man probes for IGF1, IGFBP5, IGFBP3, SFRP4, DKK2, DCN, MT2 and MMP3.

For real time quantitation of CITED1, DHH, PODXL, KRT1-19, CDH5, ENO3 and DKK3 RNAs, the Universal Probe Library system (Roche Rotkreuz, Switzerland) was used and primers were designed according to the ProbeFinder software (http://www.roche-applied-science.com).

The following primers were designed:

ENO3:

forward5′CCGGGAAATCCTGGACTC3′

reverse 5′CAGCTGCTCGGAATCGAC3′

CDH5:

forward5′TCACCTTCTGTGAGGAGATGG3′

reverse 5′GATGATCAGCAAGGTAATCACTGT3′

PODXL:

forward5′GGATCTCCCAGAGGAAGGAC3′

reverse 5′CAAGGTTGGGTTGTCATGGT3′

DKK3:

forward5′CTCAATGAGATGTTTCGAGAGGT3′

reverse 5′ CTTCTTCCGCCTCCATCTC 3′

CITED1:

forward5′CTAGGTCGCTTCGTCCGTA3′

reverse 5′AGCTGGGCCTGTTGGTCT3′

KRT1-19:

forward5′TGGAGATGCAGATTGAGAGC3′

reverse 5′TCCTCAGGGCAGTAATTTCC3′

For GAPDH amplification, rodent GAPDH control reagent (Applied Biosystems) was used. Relative quantitation of target, normalized with an endogenous control (GAPDH), was performed using a comparative Ct method (Applied Biosystems).

### IGF1 promoter analysis and cloning

In silico IGF1 promoter analysis was performed using the Genomatix (http://www.genomatix.de) genome annotation tool “Eldorado”. Specifically, GEMS analysis was used with both MatInspector and ModelInspector tools to search for putative transcription factor binding sites and sequence models [22]. Comparative genomix was also used.

The human IGF1 promoter sequence from position –1682 to +1 (start codon on exon 1) was amplified from hMSC genomic DNA using primers: 5′CCCAAGCTTGGGTGCTTCTGAAGTACAAAGTCTG3′ and 5′GAAGATCTTCAAATGTTGCTGAACATAGTGCACCATTG3′.

The amplified fragment was ligated into the BglII and HindIII cloning sites of the pGL3-basic vector (promega) and sequence-verified. Smaller IGF1 promoter fragments (−1098, −913 and –673) were obtained from the –1682pGL3 construct by digestion and re-ligated into the pGL3-basic vector. Restriction enzyme digestion was as follows: HindIII/XhoI to obtain the −1098 fragment, : HindIII/KpnI to obtain the –913 fragment and HindIII/AvaI to obtain the –673 fragment.

### Transient transfection and luciferase assays

2 µg of empty pMSCV plasmid or pMSCV encoding human FLI-1, ERG-1, EWS-FLI-1, EWS-ERG, FUS-ERG, EWS-WT1 and the EWS-FLI-1 R340N DBD mutant were introduced by nucleofection along with 2 µg of a pGL3 basic vector or the pGL3-*IGF1* promoter construct (−1682pGL3) into freshly isolated human mesenchymal stem cells using a nucleofector II device and the appropriate solution according to manufacturer's recommendation (Amaxa GmbH, Koeln, Germany). For promoter region analysis, 2 µg of pMSCV encoding human EWS-FLI-1 were introduced by nucleofection along with 2 µg of a pGL3 basic vector or one of the pGL3-*IGF1* promoter constructs. General transfection efficiency was controlled using a pMAX-GFP control vector (AMAXA) or a pGL3 promoter vector containing a SV40 promoter. A 75% efficiency was calculated in MSCs using nucleofection. Transfection efficiency for both fusion proteins and luciferase reporter plasmid was more specifically controlled by PCR or RT-PCR performed on RNA or DNA using primers specific for either luciferase or each of the fusion genes. Luciferase activity was measured 48 hours later on cleared cell lysates using the luciferase assay system (Promega) according to manufacturer's recommendations. 3 independent experiments were performed using 2 different batches of hMSCs. Reporter luciferase activity was measured in triplicate, mean values were corrected for total cell number and reported as relative values to vector transfected cells. Total protein normalization was not possible due to the presence in the luciferase kit lysis buffer of components that disrupt common total protein measurement assays. Nevertheless parallel experiments were performed with fractions of the same cell populations lysed in appropriate buffer to control the correspondence between cell number and total proteins.

Statistical significance was determined with a 2 sided t-test on the logarithm of the relative activity.

### Chromatin immunoprecipitation

ChIP was performed according to the Abcam protocol (Abcam, Cambridge, UK). Briefly 1×10^7^ mouse MPCs expressing either EWS-FLI-1, EWS-FLI-1 R340N mutant or an empty pMSCV vector, were cross-linked with 1% folmaldehyde for 10 min. After addition of 0.125 M glycine and washing in cold PBS, cells were lysed and the chromatin fraction was sheared to roughly 700bp fragments by sonication. About 1/15 of the lysate was digested with proteinase K, phenol chloroform extracted, ethanol-precipitated and stored as input DNA. Immunoprecipitation was performed using a chip-grade rabbit polyclonal anti V5 antibody (Abcam) and herring sperm DNA blocked protein A-sepharose beads. The cross linkage was reversed using proteinase K and the DNA purified by phenol/chloroform extraction and ethanol precipitation. Quantitative PCR on the immunoprecipitate and on input DNA was performed on a ABI Prism 7700 instrument (Applied Biosystems). Primers complementary to the mouse IGF1 promoter region from –2754 to –2683 and control primers spanning a region 14000 bp upstream of the IGF1 exon 1 start codon were designed using the Assay Design Center ProbeFinder (Roche) and were as follows:

Forward: 5′ TGCCTGGCAACTAGGACAA 3′

Reverse: 5′ GATCGAAAGGCAGCTCTCAG 3′

Control Forward: 5′ AGGTCCAAAAGTTGCATCAGA 3′

Control Reverse: 5′ CGAGACTCCCTGCCTTAAAA 3′

Primers complementary to murine GAPDH DNA were used as the Input control.

## Supporting Information

Figure S1 (0.38 MB TIF)

Figure S2 (0.69 MB DOC)

Table S1 (0.06 MB DOC)

Table S2 (0.81 MB XLS)

Table S3 (0.08 MB XLS)
