# Supplementary figures and images for: IGF1 Is a Common Target Gene of Ewing's Sarcoma Fusion Proteins in Mesenchymal Progenitor Cells
Source: PLoS One. 2008 Jul 9;3(7):e2634. doi: 10.1371/journal.pone.0002634 (PMC2481291; doi:10.1371/journal.pone.0002634)

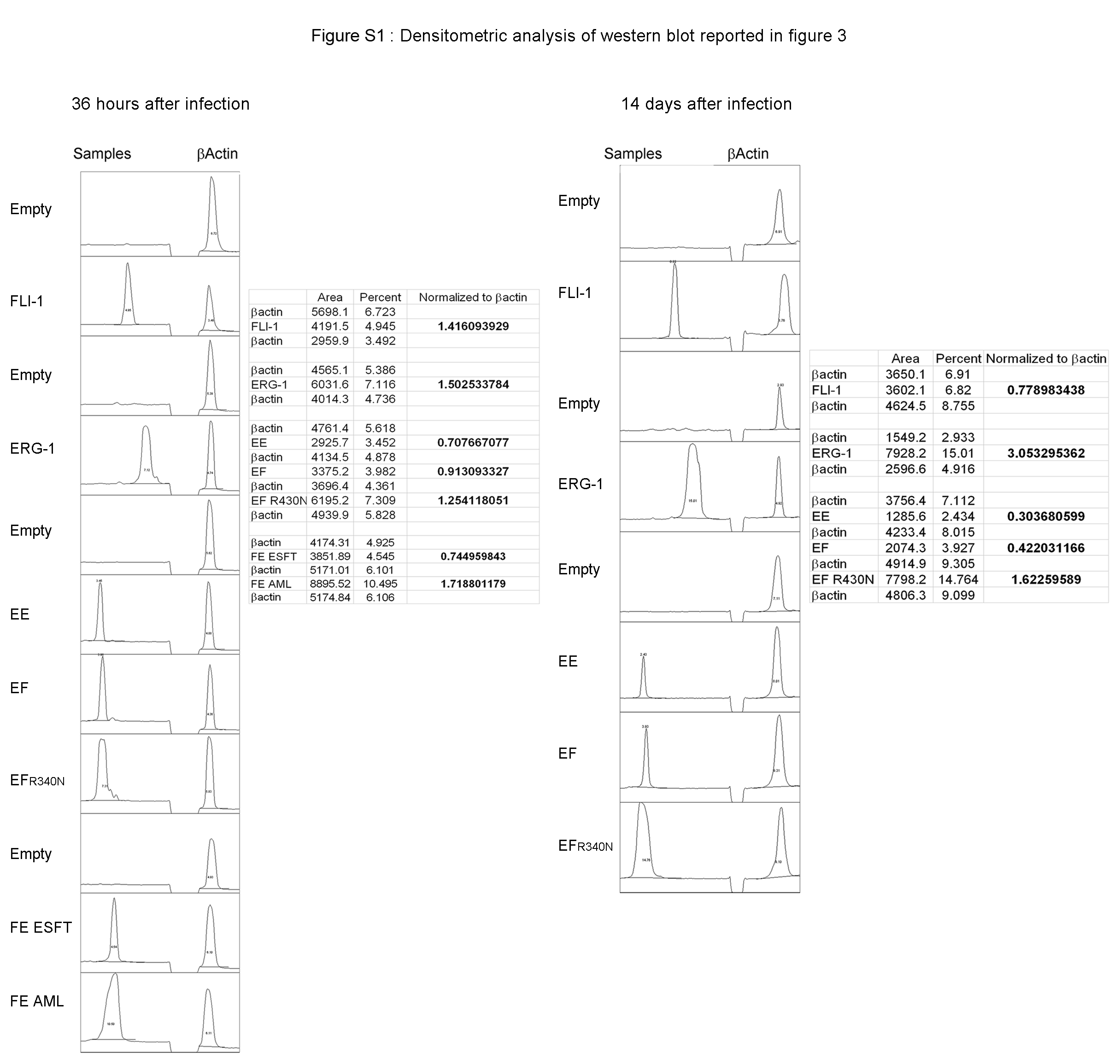

Supplement: Figure S1 — (0.38 MB TIF) [file pone.0002634.s001.tif]

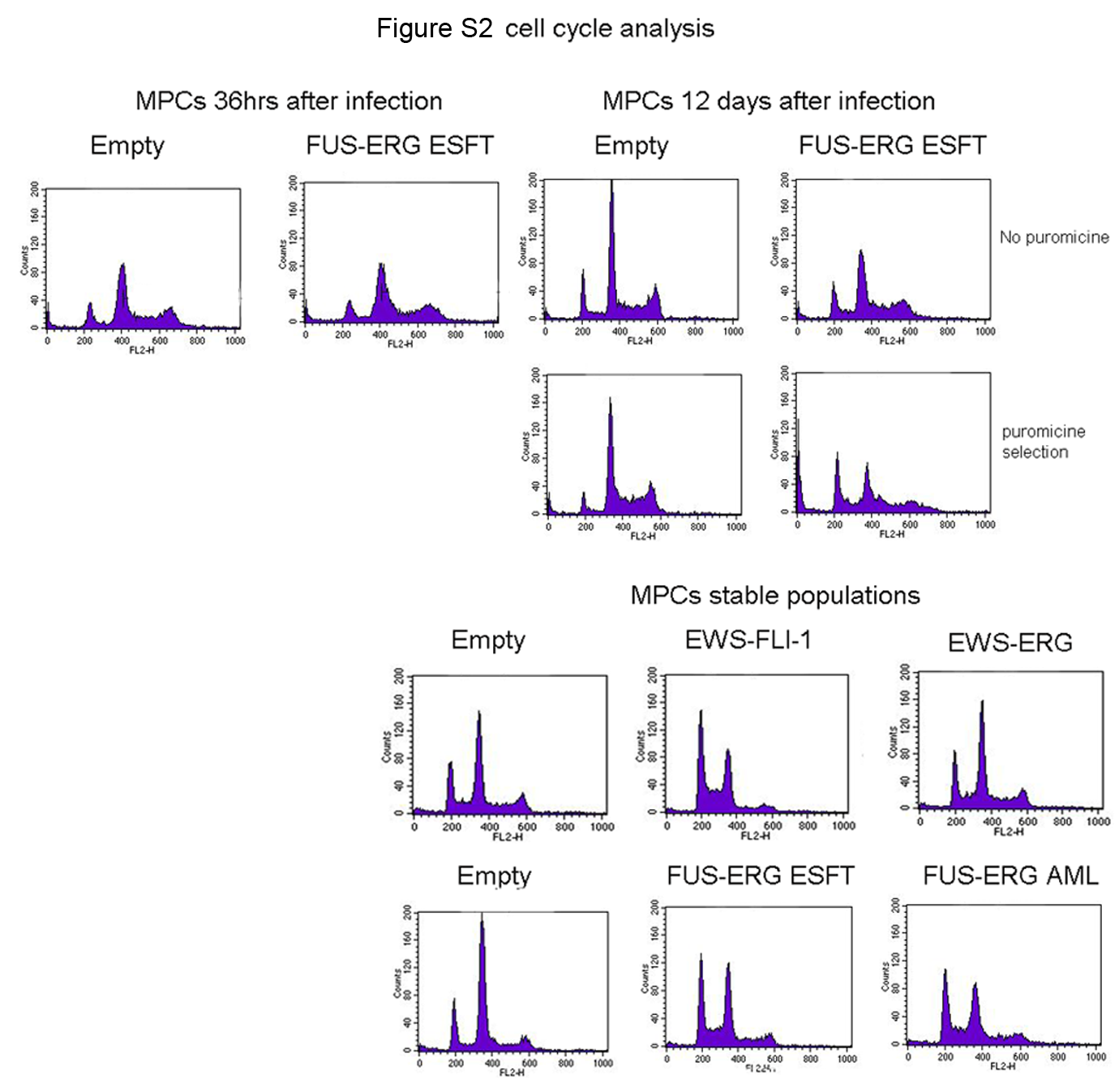

Supplement: Figure S2 — (0.69 MB DOC) [file pone.0002634.s002.tif]
