# Supplementary material for: IGF1 Is a Common Target Gene of Ewing's Sarcoma Fusion Proteins in Mesenchymal Progenitor Cells
Source: PLoS One. 2008 Jul 9;3(7):e2634. doi: 10.1371/journal.pone.0002634 (PMC2481291; doi:10.1371/journal.pone.0002634)
Supplement: Table S1 — (0.06 MB DOC) [file pone.0002634.s003.doc]

| Table S1: number of genes affected by Ewing's fusion proteins, EWS/FLI-1 R340N, wild type proteins and FUS/ERG AML fusion. | | | | | |
| --- | --- | --- | --- | --- | --- |
|
| number of repressed genes | | | number of induced genes | | |
|
|  |  | P value |  |  | P value |
| EWS/ERG | 67 |  | EWS/ERG | 144 |  |
|
| EWS/FLI-1 | 199 |  | EWS/FLI-1 | 250 |  |
|
| FUS/ERG Ewing's | 172 |  | FUS/ERG Ewing's | 208 |  |
|
|  |  |  |  |  |  |
| common to EWS/FLI-1 and EWS/ERG | 30 | 6.19E-31 | common to EWS/FLI-1 and EWS/ERG | 112 | 3.36E-149 |
|
| common to EWS/FLI-1 and FUS/ERG Ewing's | 39 | 5.28E-27 | common to EWS/FLI-1 and FUS/ERG Ewing's | 71 | 2.58E-56 |
|
| common to EWS/ERG and FUS/ERG Ewing's | 20 | 5.00E-18 | common to EWS/ERG and FUS/ERG Ewing's | 67 | 4.57E-70 |
|
| common to EWS/ERG, EWS/FLI-1 and FUS/ERG Ewing's | 13 |  | common to EWS/ERG, EWS/FLI-1 and FUS/ERG Ewing's | 57 |  |
|
|  |  |  |  |  |  |
| ERG-1 | 329 |  | ERG | 122 |  |
|
| FLI-1 | 169 |  | FLI-1 | 290 |  |
|
|  |  |  |  |  |  |
| common to ERG-1 and FLI-1 | 8 | 0.41 | common to ERG and FLI-1 | 14 | 0.00015 |
|
| common to EWS/FLI-1 and FLI-1 | 17 | 0.00000114 | common to EWS/FLI-1 and FLI-1 | 18 | 0.00504 |
|
| common to EWS/ERG and ERG-1 | 24 | 6.99E-17 | common to EWS/ERG and ERG | 18 | 5.53E-12 |
|
| common to EWS/ERG, EWS/FLI-1, FLI-1 and ERG-1 | 1 |  | common to EWS/ERG, EWS/FLI-1, FLI-1 and ERG | 4 |  |
|
|  |  |  |  |  |  |
| EWS/FLI-1 | 199 |  | EWS/FLI-1 | 250 |  |
|
| EWS/FLI-1 R340N | 167 |  | EWS/FLI-1 R340N | 98 |  |
|
| common to EWS /FLI-1 and EWS/FLI-1 R340N | 46 | 4.71E-36 | common to EWS /FLI-1 and EWS/FLI-1 R340N | 19 | 1.65E-10 |
|
|  |  |  |  |  |  |
| FUS/ERG AML | 158 |  | FUS/ERG AML | 202 |  |
|
| FUS/ERG Ewing's | 172 |  | FUS/ERG Ewing's | 208 |  |
|
| common to FUS/ERG AML and FUS/ERG Ewing's | 60 | 1.52E-61 | common to FUS/ERG AML and FUS/ERG Ewing's | 103 | 2.42E-117 |
|
